# Intradermal acupuncture for rheumatoid arthritis: study protocol for a randomised controlled trial

**DOI:** 10.1186/s13063-021-05416-0

**Published:** 2021-07-14

**Authors:** Huifang Luo, Jie Peng, Qing Ma, Zhihua Wei, Changsong Lin, Mingying Zhang, Peiwu Li, Yang Song, Xiangwei Yang

**Affiliations:** 1 School of Nursing, Guangzhou University of Chinese Medicine, Guangzhou, 510405 Guangdong Province China; 2 Department of Spleen and Stomach Diseases, The First Affiliated Hospital of Guangzhou University of Chinese Medicine, Guangzhou, 510405 Guangdong Province China

**Keywords:** Rheumatoid arthritis, Intradermal acupuncture, Protocol, Randomised controlled trial

## Abstract

**Background:**

Rheumatoid arthritis (RA) is a common autoimmune disease that severely impacts quality of life. Currently available medications for the treatment of RA have adverse side effects. Emerging evidence suggests that intradermal acupuncture (IA) is feasible and safe for patients, but its application in RA patients has not been examined. Our study aims to explore the efficacy and safety of IA for the treatment of RA.

**Methods:**

This study is a randomised, sham-controlled, patient-outcome assessor-statistician blind trial that aims to evaluate the effects of IA in patients with RA. We will recruit 132 patients aged ≥ 18 years with a diagnosis of RA. Patients will be randomly allocated with a 1:1 ratio to IA or sham IA groups. Both groups will receive basic treatment and nursing routines for RA. The experimental group will receive actual IA treatment, whereas the control group will receive sham IA treatment. All patients will receive one course of treatment (i.e., four consecutive treatment sessions with an intervening 1-day interval). Primary outcomes will be traditional Chinese medicine (TCM) syndromes before and after a treatment course and Health Assessment Questionnaire (HAQ) scores. Secondary outcomes will be disease activity score 28 (DAS28) and levels of serum C-reactive protein (CRP). Outcome measures will be collected pre- and post-treatment.

**Discussion:**

This study aims to provide high-quality evidence for the efficacy and safety of IA for treating RA. In addition, the results will provide references for selection of acupoints for other syndromes in clinical practice.

**Trial registration:**

Chinese Clinical Trial Registry ChiCTR2000038028. Registered on 8 September 2020.

**Supplementary Information:**

The online version contains supplementary material available at 10.1186/s13063-021-05416-0.

## Background

Rheumatoid arthritis (RA) belongs to the category of “Wangbi” in Chinese medicine and is a common autoimmune disease associated with chronic synovial cell proliferation and inflammatory cell infiltration [1]. “Wangbi” is a disease caused by wind, cold, damp heat, and other external forces that block meridians and collateral joints, resulting in poor blood and qi movement. The main clinical manifestations include swelling, weightiness, and pain in joints throughout the body. This condition is characterised by joint pain, swelling, morning stiffness, and functional damage, which may lead to irreversible disability and significantly impact quality of life [2]. At present, curative treatments are lacking, and patients require treatment throughout life. In recent years, several clinical indications have suggested that the treatment of RA should be a combination of pharmacotherapy and comprehensive therapeutic modalities in order to achieve the best curative effects with minimal side effects [3–5].

Intradermal acupuncture (IA) is an approach that involves insertion of needles for a prolonged period. After a needle is buried under the skin, it can produce continuous and stable stimulation of the body and promote the effective operation of meridian qi and blood flow. Unlike conventional acupuncture, IA does not require lifting, pulling, or other actions and is therefore highly convenient.

IA has the advantage of providing patients with continuous stimulation and treatment without affecting daily activities, particularly for cases of chronic intractable pain. Moreover, this approach involves a combination of acupuncture and exercise therapy, which promotes qi and blood circulation, dredging meridians, and metabolism. As needles are restricted to the subcutaneous layer and do not penetrate the deep layer, the viscera, nerve trunk, and large blood vessels are preserved, and the technique is safe. IA causes minimal pain given that short needles are used [6]. Further, dizziness occurs less frequently in IA than in traditional acupuncture [7, 8]; hence, IA is more acceptable to patients.

Although IA is a new technology, there are still some other countries except China, such as Korea [9–11] and Japan [12], that have used IA treatment in the clinical practice. IA treatment is considered feasible and safe for patients with advanced cancer pain [9], post-stroke patients [6], and primary dysmenorrhea patients [13]. However, there is a paucity of reports on the application of IA in patients with RA. This study aims to investigate the clinical efficacy, adverse reactions, and safety of IA as a treatment for RA in order to promote clinical application of IA, accelerate disease rehabilitation, and improve patients’ quality of life.

## Methods/design

### Aim

The aim of this study will be to design a detailed research protocol for IA treatment for RA patients in China and to explore its clinical efficacy, adverse reactions, and safety.

### Study design

**Fig. 1:**
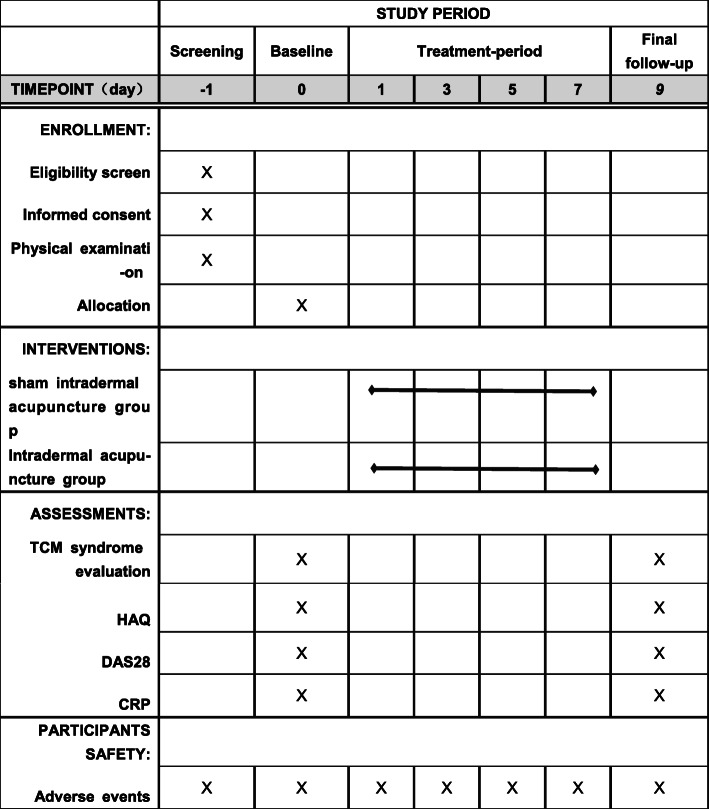
The schedules for patient enrollment, interventions, and assessments. **TCM**, traditional Chinese medicine; **HAQ**, Health Assessment Questionnaire; **DAS28**, disease activity score 28; **CRP**, C-reactive protein

**Fig. 2:**
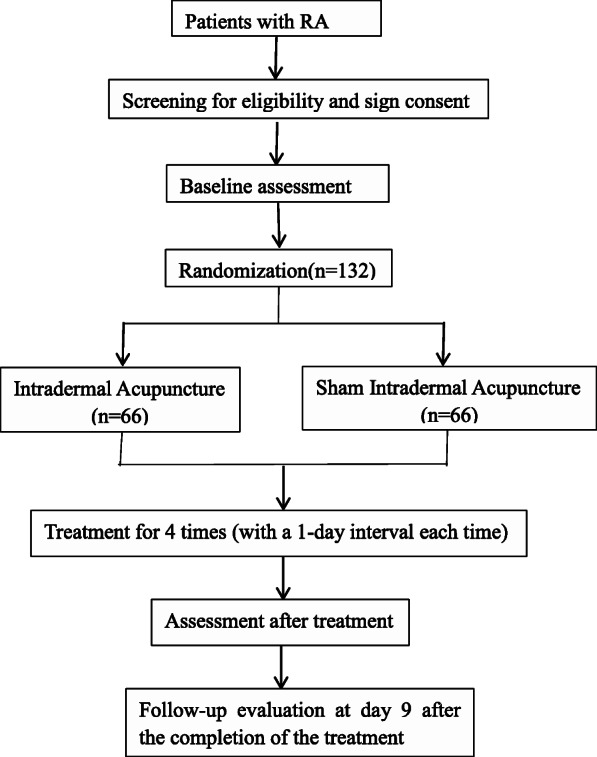
Flowchart of the study design

This is a two-arm, single-centre, randomised controlled trial conforming to the Standard Protocol Items: Recommendations for Interventional Trials (SPIRIT) guidelines (Additional file and Fig. 1). The process of participant inclusion is illustrated in Fig. 2.

### Participants

Patients will be recruited by trained nurses from the Department of Rheumatology at a specific first-class hospital in Guangdong. More than 500 patients are admitted annually, ensuring that a sufficient number of clinical research cases can be included in the trial. All patients will undergo a general physical examination including electrocardiogram (ECG), chest film, routine blood work, examination of liver and kidney function, assessment of vital signs (body temperature, pulse, breathing, and blood pressure), examination of serum C-reactive protein (CRP) levels, coagulation function test, and disease activity score 28 (DAS28) prior to inclusion.

### Recruitment strategy

Patients will be recruited from the inpatient Department of Rheumatology at a specific first-class hospital in Guangdong from December 2020 to March 2022. Recruitment posters will be posted at the hospital. Two researchers will assess patient eligibility and provide study information, including study objectives, intervention measures, treatment time, benefits, and potential risks. Patients who agree to participate in this study will sign an informed consent form.

### Diagnostic criteria

The diagnostic criteria comprise Western and traditional Chinese medicine (TCM) diagnostic criteria. The Western diagnostic criteria are based on the 1987 American Rheumatology Association (ARA) revised RA classification criteria [14] and the 2009 American College of Rheumatology (ACR)/European League Against Rheumatism (EULAR) RA classification criteria [15]. TCM diagnostic criteria are based on the Chinese medicine industry standard of the People’s Republic of China (diagnostic criteria of TCM syndromes) (ZY/T001.1-94).

### Inclusion criteria

Patients will be included if they meet the following five criteria: (1) match the diagnostic criteria of RA, (2) meet the reference of “State Administration of TCM ‘Eleventh Five-Year’ key specialised cooperative group Wangbi (RA) diagnosis and treatment plan” syndrome indicative of liver and kidney deficiency, (3) DAS28 score of “mild” or “inactivity” with concomitant arthropathy in the extremities, (4) age ≥ 18 years, and (5) a signed informed consent form.

### Exclusion criteria

Patients will be excluded if they meet any of the following criteria: (1) present with comorbid diseases, including severe cardiopulmonary disease, liver or kidney disease, coronary heart disease, diabetes, blood diseases, or malignant tumours; (2) pregnant or lactating women; (3) fever or clotting disorder; (4) receiving treatment for local skin ulcers or skin diseases; (5) allergic constitution and allergies to adhesive tape; and (6) participation in any other clinical trials.

### Power calculation

Sample size was determined based on the formula for sample size calculation comparing two groups with independent sampling rates:

$$ \mathrm{n}1=\mathrm{n}2=\frac{{\left({Z}_{1-\alpha /2}+{Z}_{1-\beta}\right)}^2\left[{P}_1\left(1-{p}_1\right)+{P}_2\left(1-{p}_2\right)\right]}{{\left({p}_1-{p}_2\right)}^2} $$

Based on previous clinical research, the rate for the trial group that received IA for RA treatment was 95% and that of the control group was 78% [16]. In this study, assuming *α* = 0.05 (both sides) and 1 − *β* = 0.80, the required sample size was calculated as 60 per group. Considering a dropout rate of 10%, we concluded that the required sample size was 66 per group. Therefore, a total of 132 patients with RA will be recruited.

### Randomisation

RA patients who meet the inclusion criteria and agree to participate in this research will be randomly allocated to either the intervention group or control group, with an allocation ratio of 1:1. The random allocation sequence will be generated by a statistician (not involved in the study) using a computer programme. Each random number with a group assignment will be written on a piece of paper and inserted into a sealed envelope. Following baseline assessment, participants will be provided with an envelope according to the randomisation sequence.

### Blinding

Blinding IA operators in acupuncture clinical trials is challenging. However, it is feasible to conceal patient and group assignments from outcome assessors and statisticians. Patients will be informed that they will receive one of two effective IA interventions after enrolment. During the IA treatment, patients in different groups will be treated in separate rooms to avoid communication. At the end of follow-up, the patients will be asked to guess which treatment they were receiving to determine the credibility of blinding. Outcome assessors and statisticians will be blinded to group allocations. Blinding will be disclosed only when it must be known whether a patient is receiving intradermal acupuncture treatment or sham acupuncture treatment in order to manage a serious adverse event.

### Interventions

**Fig. 3:**
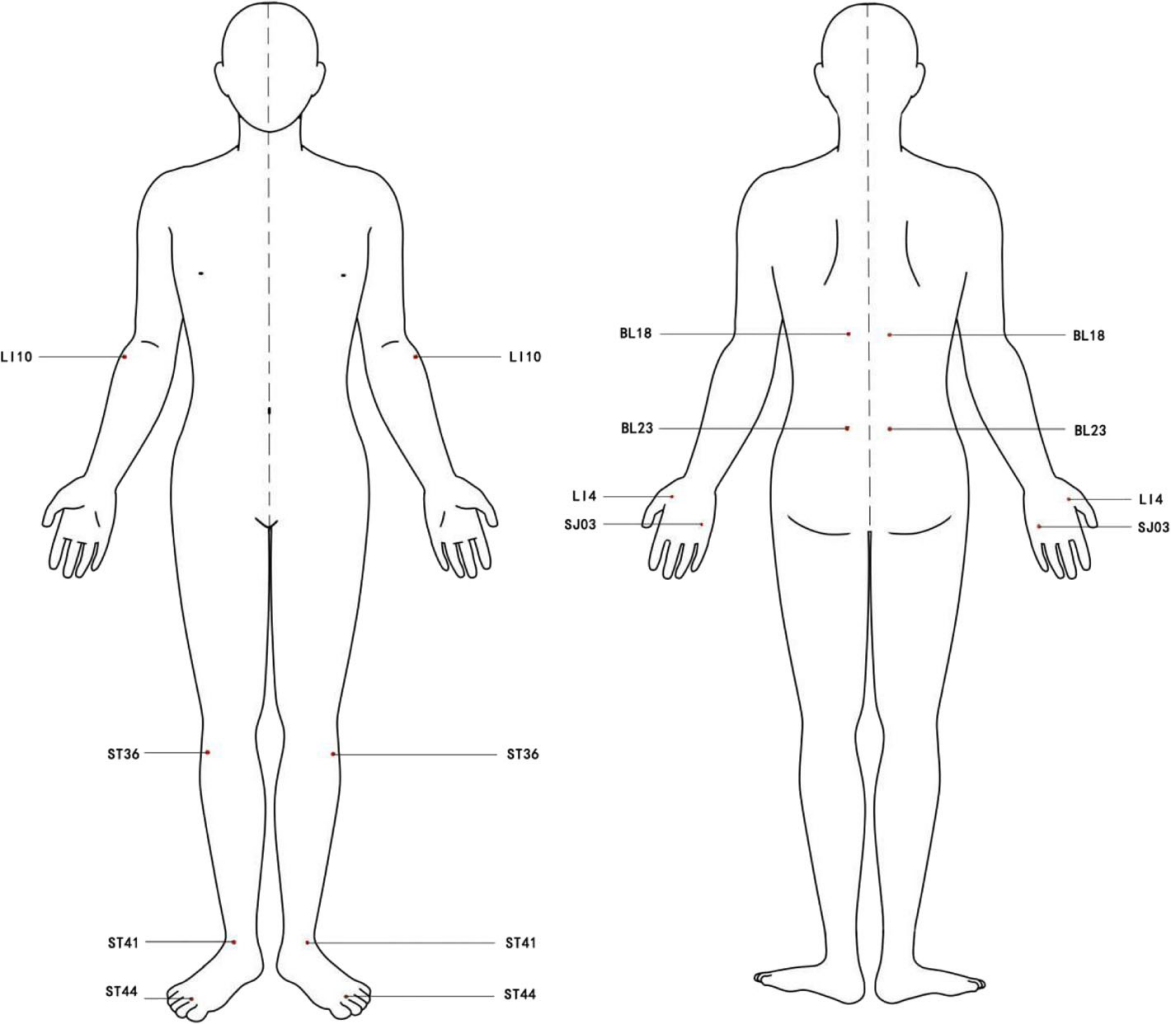
Locations of acupoints. LI10 (Shou Sanli), 3 cun below the lateral epicondyle of the humerus. ST36 (Zu Sanli), located on the outside of the calf, 3 in. below the calf’s nose. ST41 (Jiexi), in the central depression of the horizontal stripes at the junction of the instep and the calf. ST44 (Neiting), seam between the second and third toes on the dorsum of the foot. BL18 (Ganshu), at the same level as the inferior border of the spinous process of the 9th thoracic vertebra (T9), 1.5 cun lateral to the posterior median line. BL23 (Shenshu), at the same level as the inferior border of the spinous process of the 2nd lumbar vertebra (L2), 1.5 cun lateral to the posterior median line. LI4 (Hegu), on the back of the hand, between the first and second metacarpal bones, the midpoint of the radial side of the second metacarpal bone. SJ03 (Zhongzhu), located on the back of the hand, 2 cm below the base of the little finger and the ring finger, the depression on the back of the hand

Patients in both groups will receive five to eight IA sessions (at least five sessions) for each treatment. After insertion, needles will be left in place for 24 h with a 1-day interval between each session. A full treatment course will constitute four sessions. Acupoint prescription will comprise acupuncture points with three needles for the hands or three needles for the feet combined with nourishing liver and kidney acupoints. Based on the location of joint pain in the limbs, the acupoints of patient with upper limb pain will be treated with three needles combined with tonification of the liver and kidney to consolidate the roots, including Shousanli, Hegu, Zhongzhu, Ganshu, and Shenshu. In patients with lower limb pain, Zusanli, Jiexi, Neiting, Ganshu, and Shenshu will be selected as acupoints for tonification of the liver and kidney (Fig. 3). During the needle retention period, other treatments will not be performed at the acupoints where the needle is retained, and other non-needle locations will not be affected.

### Control intervention

RA patients will receive basic treatment and nursing routines. Basic treatment will comprise oral celecoxib, leflunomide, and methotrexate. Routine nursing measures will encompass lifestyle, diet, medication, psychology, and exercise. Patients will be required to remain vigilant regarding joint warmth and avoid cold stimulation and/or long-term weight-bearing of small joints. Patients will be encouraged to adopt a healthy diet and lifestyle by prioritising nutritious low-salt and low-fat foods, abstaining from fried or spicy food, and quitting smoking and alcohol. Nursing staff will introduce commonly used drugs, basic medication methods, and basic pharmacological effects of clinical drugs to improve patients’ understanding of their daily medication. Patients will be encouraged to stay positive and maintain social interactions. Patients will be encouraged to perform slow or fast walking, square dancing, joint exercises, and other activities, as desired.

The aforementioned intervention acupoints will be treated with pseudo-intradermal needling (placebo). No needles will be present under the adhesive tape of the sham treatment group; the tape will merely be adhered to the surface of acupoints. Patients will be required to press the needle embedding points for 1 to 2 min every 3 to 4 h to simulate an increase in stimulation and efficacy.

### Experimental intervention

Measures for the experimental group will be similar to those for the control group, as described above, with the exception that the experimental group will receive actual needle treatment rather than sham treatment. Acupuncture needles (0.2-mm diameter and 1.2-mm length; Qingling, Japan) will be provided by Sichuan Yuanquan Medical Instrument Co., Ltd., China. The skin will first be disinfected with 75% alcohol. Sealing paper will be removed from an acupuncture needle. Next, the plastic container will be twisted backward. Half of the peeling paper and tape will be clamped with the thumb and forefinger, followed by separation from the other half of the paper and removal of the needle from the plastic container. The needle will be applied directly to the sterilised acupuncture point and inserted. The release paper will be removed, and the tape will be pressed to ensure secure adhesion. Each acupuncture point will be treated according to this procedure. Needles will be removed after 24 h. Patients will be required to press the needle embedding points for 1 to 2 min every 3 to 4 h to promote stimulation and enhance treatment efficacy.

### Outcomes

#### Primary outcomes

Primary outcomes are the evaluation of TCM syndromes before and after a course of treatment and Health Assessment Questionnaire (HAQ) scores.

TCM syndrome evaluation will be conducted according to the 2002 “Guiding Principles for Clinical Research of New Chinese Medicines [17]” based on the degree of joint pain; number of joints involved; joint swelling, tenderness, flexion, and extension; and other symptoms or signs. Scores will be divided into four levels: none, 0 points; light, 1 point; severe, 2 points; and very severe, 3 points. Four types of curative effects will be considered: (1) recovery: TCM syndrome scores decreased by ≥ 70%; (2) significant effect: TCM syndrome scores decreased by ≥ 50%; (3) effective: TCM syndrome scores decreased by ≥ 20%; and (4) ineffective: TCM syndrome scores decreased by less than 20%, whereby effectiveness rate = ([points before treatment – points after treatment]/points before treatment) × 100%.

HAQ scores will be used to assess patients’ physical function [18]. The HAQ is a widely used 20-item questionnaire that encompasses the following dimensions: dressing, washing, standing up, walking, and other aspects of daily life. Each question is scored on a scale from 0 to 3 points (0, no difficulty; 1, some difficulty; 2, very difficult; 3, unable to complete). The total score ranges from 0 to 60 points, and average scores range between 1 and 3 points. Higher scores indicate poorer health status.

#### Secondary outcomes

Secondary outcomes are DAS28 and CRP levels. For DAS28, general health (GH), 28-joint swelling index (SJC), joint tenderness count (TJC), erythrocyte sedimentation rate (ESR), and visual analogue scale (VAS) scores will be evaluated. The 28 joints include the shoulder, elbow, wrist, metacarpophalangeal, proximal interphalangeal, and knee joints. The formula is as follows [19]:

$$ \mathrm{DAS}28-\mathrm{ESR}=0.56\times \sqrt{\left(\mathrm{TJC}28\right)}+0.28\times \sqrt{\left(\mathrm{SJC}28\right)}+0.70\times \ln \left(\mathrm{ESR}\right)+0.014\times \mathrm{GH}. $$

The level of disease activity will be categorised as follows: remission (≤2.6), low activity (>2.6 and ≤3.2), moderate activity (>3.2 and ≤5.1), and high activity (>5.1).

CRP is a non-specific marker of systemic inflammation that directly reflects the degree of inflammation and infection. CRP is widely used in clinical diagnosis and assessment of treatment efficacy for rheumatic and autoimmune diseases, especially during the active stage of the disease. Higher serum CRP levels indicate greater disease severity.

### Patient safety

To guarantee patient safety, acupuncture treatment will be performed in the rheumatology department to ensure that rheumatologists and first-line physicians will be able to respond to emergencies in a timely manner. Adverse events such as pain, dizziness, pruritus, and local redness will be carefully recorded in case report forms (CRFs). Serious adverse events such as death or life-threatening events will be immediately reported to the principal investigator.

### Practitioner training and quality control

Practitioners and press needle operators will be required to undergo rigorous training and consistency testing to ensure consistency in comprehension and implementation of interventions. All acupuncture manipulations will be performed by two licensed head nurses with at least 5 years of TCM clinical experience. Two team members will inspect measurements during progress. Data analysts will not participate in clinical implementation and project design to ensure appropriate blinding.

### Data management

Data will be recorded using printed CRFs. Relevant electronic records will be assessed and entered by two team members. Only outcome assessors will have access to CRFs and will perform data entry. When patients suffer death or life-threatening serious adverse events, our researchers will immediately report to main investigators, although serious adverse events will not be anticipated. The main investigators will make the final decision to terminate the trial after comprehensive discussion.

The data monitoring committee of the Rheumatology Department in the First Affiliated Hospital of Guangzhou University of Chinese Medicine which is independent from the funder and any competing interests will set up to monitor regularly the performance and safety of the trial. It is composed of independent clinical experts and statisticians. They will check the randomly assigned participants meet the inclusion and exclusion criteria, ensure the trial is proceeding smoothly, and inspect adequate data is recorded in the CRFs. There will be no interim analysis. This trial will continue until the 132 participants have completed this trial.

### Adherence improvement

Before the participants are enrolled in the study, our researchers will inform the patient of the study information, including study objectives, intervention measures, treatment time, benefits, and potential risks. These lay the foundation for patient compliance. During the intervention period, our researchers will instruct patients to press the needle embedding points for 1 to 2 min every 3 to 4 h to simulate an increase in stimulation and efficacy. Two team members will also inspect measurements during progress for monitoring adherence. The data monitoring committee will also inspect data is adequately recorded in the CRFs. If patients suffer an adverse event, our rheumatologists and first-line physicians will respond to emergencies in a timely manner and provide free medical guidance. After all follow-ups, we will provide 3 months of free intradermal acupuncture for patients in the sham group. All treatment and examination costs in this study are free and borne by the team. Certain transportation subsidies will also be provided. These will contribute to improving adherence.

### Confidentiality

All researchers will sign a confidentiality agreement to protect the privacy of participants. Patient’s medical records and CRFs are kept in the hospital as required and they will be retrieved when necessary. Patient’s information and illness will not be discussed in public. Patient’s personal information will not be provided to other institutions or individuals. Besides, data will be anonymised prior to publication to prevent identification of individual participants.

### Data analysis

Data analysis will be performed using SPSS software (version 23.0; IBM SPSS Statistics, New York, USA) by statisticians independent of the research team.

Baseline data including age, sex, disease duration, evaluation of TCM syndromes, HAQ score, DAS28 score, and CRP values before interventions will be compared between the two groups. All analyses will be strictly conducted according to the intention-to-treat principle. Continuous variables such as age, disease course, and values of DAS28, HAQ, and CRP will be presented as mean ± standard deviation (SD). Categorical variables such as TCM syndromes will be presented as percentages. Student’s *t* tests will be used to compare quantitative data between the two groups. Data on adverse reactions will be analysed using the *X*^2^ test. Differences will be considered statistically significant at *P* < 0.05. Missing data will be imputed by multiple imputation. To assess the robustness of the results, a sensitivity analysis using a pattern-mixture model will be used.

## Discussion

RA is a chronic autoimmune disease which presents with serious disability and dysfunction [5, 20]. RA affects 0.5% of adults worldwide and is two to three times more common in women than in men [21]. The incidence of RA in the USA and China is 0.5–1.0% [2] and 1.02%, respectively [22]. DAS28-CRP is widely used to monitor disease activity and is based on the number of swollen or tender joints, serum CRP, and global patient assessment [23, 24]. Pharmacotherapy is the first line of treatment for RA. The application of anti-rheumatic drugs at early diagnosis and treatment of RA improves joint swelling and tenderness and delays the process of joint destruction [25, 26]. Nevertheless, adverse side effects of drugs are common. Therefore, there has been growing interest in complementary and alternative medicine treatments for RA.

Acupuncture is an important component of TCM and is widely used in the treatment of patients with RA in China. Evidence suggests that acupuncture alone or in combination with other treatment modalities is beneficial for clinical treatment of RA, does not result in adverse effects, and significantly improves quality of life and function [5, 27]. Nevertheless, traditional acupuncture has drawbacks. For instance, patients may be apprehensive about undergoing continuous traditional acupuncture therapy due to time and manipulation factors. To address this, IA may be selected. Intradermal needles were developed using the ancient shallow needling method. These small needles are inserted into the subcutaneous tissue and are retained after fixation. This enables continuous stimulation of the body over a prolonged period, a state termed “embedding needles”, which combines the advantages of embedding thread and acupuncture. IA has the advantages of simple operation, lasting effects, low pain stimulation, and high compliance. It is particularly suitable for individuals who are apprehensive about acupuncture [28].

In this study, the inclusion criterion of age was set to above 18 years to ensure coverage of a wide age range. In addition, we will enrol RA patients with liver and kidney deficiency syndrome as research participants for third reasons. First, TCM nursing technology places substantial importance on the principle of “syndrome differentiation and treatment” via syndrome differentiation and acupoint selection. In this regard, the optimal therapeutic effects can be achieved and observed more objectively and comprehensively. Second, liver and kidney deficiency syndrome is a common syndrome observed in clinical practice [29]. Third, RA pathogenesis is underpinned by a deficiency in healthy qi and pathogenic factors. Treatment should therefore focus on eliminating pathogenic factors and strengthening healthy qi. Selecting patients with liver and kidney deficiency syndrome will help to strengthen the body and achieve better therapeutic effects.

A limitation of our trial is that IA operators will not be blinded to the nature of the intervention. To reduce confounding effects on patients, operators will be informed of patient group assignments just before treatment. The results of this trial will provide more reliable evidence and clarify the value of IA as a treatment for RA. Further, our study will provide a reference for selecting acupoints for other syndromes.

## Trial status

This trial was registered on 8 September 2020 at the Chinese Clinical Trial Registry (Registration number: ChiCTR2000038028, the protocol version number: 2.0, version date: 19 August 2020). This trial is currently in the stage of patient recruitment. The first patient was enrolled on 15 December 2020. Patient recruitment is expected to end on 30 March 2022.

## Supplementary Information

**Additional file 1.** SPIRIT 2013 Checklist: Recommended items to address in a clinical trial protocol and related documents*.

## Data Availability

This trial does not involve the storage of biological specimens. The data and materials used during the current study are available from the corresponding author on reasonable request.

## Abbreviations

IA: Intradermal acupuncture
RA: Rheumatoid arthritis
TCM: Traditional Chinese medicine
ECG: Electrocardiogram
ARA: American Rheumatology Association
ACR: American College of Rheumatology
EULAR: European League Against Rheumatism
HAQ: Health Assessment Questionnaire
DAS28: Disease Activity Score 28
CRP: Serum C-reactive protein
GH: General health
SJC: 28 Joint swelling index
TJC: Joint tenderness count
ESR: Erythrocyte sedimentation rate
VAS: Visual analogue scale
CRFs: Case report forms
