# Correction to: Intradermal acupuncture for rheumatoid arthritis: study protocol for a randomised controlled trial

**DOI:** 10.1186/s13063-021-05514-z

**Published:** 2021-08-12

**Authors:** Huifang Luo, Jie Peng, Qing Ma, Zhihua Wei, Changsong Lin, Mingying Zhang, Peiwu Li, Yang Song, Xiangwei Yang

**Affiliations:** 1 School of Nursing, Guangzhou University of Chinese Medicine, Guangzhou, 510405 Guangdong Province China; 2 Department of Spleen and Stomach Diseases, The First Affiliated Hospital of Guangzhou University of Chinese Medicine, Guangzhou, 510405 Guangdong Province China

**Correction to: Trials 22, 450 (2021)**

**https://doi.org/10.1186/s13063-021-05416-0**

Following the publication of the original article [1], the authors notified us that the affiliation for Peiwu Li should remain the Department of Spleen and Stomach Diseases... (affiliation number 2). However, this affiliation should be removed for for Mingying Zhang, Changsong Lin and Xiangwei Yang, as it was mistakenly added during proofs. The correct affiliation for Mingying Zhang, Changsong Lin and Xiangwei Yang should be affiliation number 1.

The original article has been corrected.
